# Variation in Older Adult Characteristics by Residence Type and Use of Home- and Community-Based Services

**DOI:** 10.3390/ijerph14030330

**Published:** 2017-03-22

**Authors:** Heidi H. Ewen, Tiffany R. Washington, Kerstin G. Emerson, Andrew T. Carswell, Matthew Lee Smith

**Affiliations:** 1 Institute of Gerontology, College of Public Health, The University of Georgia, 102 Spear Road, Hudson Hall, Athens, GA 30602, USA; emerson@uga.edu (K.G.E.); health@uga.edu (M.L.S.); 2 Department of Financial Planning, Housing, and Consumer Economics, The University of Georgia, Athens, GA 30602, USA; carswell@uga.edu; 3 Department of Health Promotion and Behavior, College of Public Health, The University of Georgia, Athens, GA 30602, USA; 4 School of Social Work, The University of Georgia, Athens, GA 30602, USA; twashing@uga.edu; 5 Department of Health Policy and Management, College of Public Health, The University of Georgia, Athens, GA 30602, USA; 6 Department of Health Promotion and Community Health Sciences, School of Public Health, Texas A&M University, College Station, TX 77843, USA

**Keywords:** aging, health, housing, service utilization, aging-in-place

## Abstract

*Background:* The majority of older adults prefer to remain in their homes, or to “age-in-place.” To accomplish this goal, many older adults will rely upon home- and community-based services (HCBS) for support. However, the availability and accessibility of HCBS may differ based on whether the older adult lives in the community or in a senior housing apartment facility. *Methods:* This paper reports findings from the Pathways to Life Quality study of residential change and stability among seniors in upstate New York. Data were analyzed from 663 older adults living in one of three housing types: service-rich facilities, service-poor facilities, and community-dwelling in single-family homes. A multinomial logistic regression model was used to examine factors associated with residence type. A linear regression model was fitted to examine factors associated with HCBS utilization. *Results*: When compared to community-dwelling older adults, those residing in service-rich and service-poor facilities were more likely to be older, report more activity limitations, and provide less instrumental assistance to others. Those in service-poor facilities were more likely to have poorer mental health and lower perceived purpose in life. The three leading HCBS utilized were senior centers (20%), homemaker services (19%), and transportation services (18%). More HCBS utilization was associated with participants who resided in service-poor housing, were older, were female, and had more activity limitations. More HCBS utilization was also associated with those who received instrumental support, had higher perceived purpose in life, and poorer mental health. *Conclusions*: Findings suggest that older adults’ residential environment is associated with their health status and HCBS utilization. Building upon the Person–Environment Fit theories, dedicated efforts are needed to introduce and expand upon existing HCBS available to facility residents to address physical and mental health needs as well as facilitate aging-in-place.

## 1. Introduction

Older adults prefer to remain in their homes, or to “age-in-place”, because doing so allows them to maintain independence [1,2]. Housing type preferences among older adults in the United States are diverse; they often vary based on financial or healthcare-related needs, and affect aging-in-place. The majority of older adults express desire to remain in their homes [3,4], a smaller proportion with fewer needs prefer to reside in independent living apartments or active living communities, and those with additional needs reside in assisted living facilities or affordable housing.

Home- and community-based services (HCBS) can facilitate the ability of older adults to remain in their own homes by providing various forms of assistance to accomplish activities of daily living [5,6]. Typical HCBS include assistance with bathing, meal provisions, homemakers, respite care, transportation, in-home health care (e.g., nurse visits, physical therapy), and legal advice [7]. However, older adults often encounter barriers to HCBS utilization [8,9], which include affordability and cost, lack of awareness, and unavailability of services. These impediments suggest the presence of an unmet need among older adults, and researchers are beginning to examine HCBS utilization patterns to increase service access and uptake among older adults who could benefit from such resources [8,9].

Barriers to utilization are problematic, because the increased burden of chronic conditions experienced by older adults further contributes to the need for HCBS. Approximately two-thirds of older adults have two or more chronic conditions [10]. The most commonly-occurring conditions include arthritis, heart disease, cancer, diabetes, and hypertension [11,12], all of which increase the risk for functional decline, impairment [13], and the need for assistance with activities of daily living (ADLs (Activities of Daily Living); e.g., feeding, dressing, bathing). This ADL assistance often becomes the responsibility of family members, most often the spouse or adult children [14,15]. In the absence of supportive services, such caregiving tasks become particularly burdensome on the older adult and care provider alike [16,17,18,19].

The physical (built) environment has been identified as an important contributor to HCBS use [20,21,22]. For example, HCBS use varies substantially across housing types based on service availability, accessibility, and geospatial proximity. While facilities such as Continuing Care Retirement Communities (CCRCs) enmesh services and housing [3,23,24]. HCBS are rarely offered accommodations in government subsidized senior housing (i.e., Section 202 housing) that provide independent living apartments to older adults of limited means [25,26].

Less is known about the influence of the social environment on HCBS use. Social isolation has adverse effects on the health and well-being of older adults, including a greater risk of disability and mortality in comparison to other age groups [27,28,29]. HCBS use can be of particular benefit to address the needs of older adults at increased risk of social isolation who have small social networks to assist them (e.g., physically, socially, financially) [30]. While substantial arguments have been made to link research about physical and social environments, research that adequately combines these aspects of aging has not proliferated [31,32].

Therefore, the purposes of the study were to (1) identify the demographics, health status, psychosocial factors, and HCBS utilization among older adults based on the type of housing in which they live; and (2) examine how demographics, health status, psychosocial factors, and residence type are associated with HCBS utilization. These purposes are achieved through the examination of three older adult subgroups who live in single-family homes (e.g., community-dwelling), service-rich facilities (e.g., CCRC), and service-poor senior housing (e.g., apartments with few to no service provisions).

### Conceptual Framework

The study of social and physical environments on the functional and psychosocial well-being of older adults is often difficult given the division of theories by discipline [20,31,33,34]. It has been proposed that the differential concepts of social and physical aspects of environment entail issues of meso- and micro-levels of analyses, thus limiting the inclusion of these aspects as covariates in the same study [31]. However, these two aspects are components within the person–environment fit processes, which considers the physical and social environments as transactional [35]. Both cross-sectional and longitudinal studies report the influence of social networks on subjective well-being, including exchanges of support [36], social contact improving mood [35,37], and adaptation to losses associated with aging [38].

This study draws upon the Lawton’s ecological model [20,34,39] and Person-Environment (P-E) framework [21]. P-E fit conceptualizes the relationships between older adults and their surrounding systems. The “person” component is comprised of individual-level competencies, such as motor skills, cognitive function, and biological health [40]. The environment is often conceptualized as the physical surroundings such as the home or community. The ecological model provides context for P-E fit through the inclusion of physical, personal, small-group (e.g., family and friends), supra-personal (e.g., proximal family/staff) and mega-social environments (i.e., culture, society) [20]. Activities of Daily Living (ADLs) limitations are an index of the relationship between the person (ability) and environment (management of tasks) [41,42]. It is hypothesized that older adults experiencing difficulties in maintaining ADL tasks may particularly benefit from assistance provided by HCBS. We use these theoretical frameworks to guide our investigation about the associations of the physical and social environments with the use of HCBS.

## 2. Materials and Methods

### 2.1. Sample

This study examined data from the Pathways to Life Quality Study, a longitudinal study of residential change and stability in an upstate New York community [43,44,45,46,47,48,49]. Samples were recruited through two pathways. Survey Sampling, Inc. and voter registration records provided the initial list of 55,000 potential participants over the age of 60 years. Using a random number generator (www.randomizer.org), names were selected at random and information about the study was mailed to them. Follow-up telephone calls were conducted a week later, and interviews were scheduled by the researchers. Letters returned with no forwarding address were flagged in the database along with any returns indicating that the contacts were deceased. The final response rate for the random sample was 43%. Convenience samples were recruited from the senior housing communities through fliers, mailings, social events and presentations. Based on these procedures, the resulting analytic sample consisted of randomly-selected community-dwelling county residents aged 60 and older (*n* = 343), residents of service-rich facilities (*n* = 184), and residents of service-poor facilities (*n* = 136). Service-rich facilities were those in which onsite services were available and included in the monthly fees. Within this study, there were two service-rich facilities. One was a continuing care retirement community (CCRC) that provided assisted and skilled-nursing levels of care, meal provisions; a rehabilitation and fitness center staffed with physical, occupational, and speech therapists; a care clinic with pharmacy; and transportation. The second service-rich facility had independent and assisted living options, a recreation suite and a partnership with a nearby college that allowed allied health students experience and credit by providing therapies to residents; a swimming pool, fitness center, and exercise classes; and transportation. Service-poor facilities included low-income HUD-subsidized apartment buildings and affordable apartments for senior living. Service provisions were not part of the housing package, but were available through Medicare/Medicaid waivers and local home health care agencies. 

The Pathways to Life Quality Study was approved by the Institutional Review Board at Cornell University and Ithaca College during data collection. Institutional Review Board (00003614) approval was granted for this secondary data analyses at the University of Georgia.

### 2.2. Measures

As the guiding theoretical framework of this study, the Ecological Model was used to inform variable selection for this study [20]. In particular, the psychosocial variables represent the small group and supra-personal aspects of the social environment. Descriptions of study variables are provided below.

#### 2.2.1. HCBS Use 

Our main variable of interest to examine across housing types was HCBS service use. The survey directly asked participants if they used HCBS. A list of services was presented, dichotomously coded as “uses” and “does not use.” HCBS included in the list were home health care, senior centers, transportation, home-delivered meals, legal assistance, and homemaker. Within this upstate, New York county home health care included visits from nurses, nurse aids, and physical or occupational therapists from area health care agencies. Senior centers provide activities, recreation, and community meals. Senior centers also assist with coordination and delivery of home delivered meals for those unable to visit the center. Legal assistance was a local service that provided assistance with wills and end-of-life documents for older adults. Homemaker services included assistance with companionship, cleaning, and errands. These variables were used in descriptive statistics and then summed into a variable of total number of services used.

#### 2.2.2. Health Status

Subjective assessments of health were ascertained through self-report of health on a ten-point ladder where 10 represented best possible health [50], a four-point scale comparing one’s own health to others one’s own age, self-report levels of pep or energy on a ten-point ladder, and summed score of ADL limitations using the Medical Outcomes Survey (MOS) scale [51]. Objective measures of health included (a) months since last physician visit; (b) incidence of hospitalization in the preceding two years; (c) the number of hospitalizations; and (d) days per hospitalization.

#### 2.2.3. Psychosocial Factors

Embedded within the interviews were scales assessing psychosocial well-being, including social integration and social support, positive and negative affect, purpose in life, instrumental support, and a one-item life satisfaction measure. The Cutrona Provisions of social relationships subscales were included as indices of psychosocial well-being, to measure social integration and social support [52]. The Positive and Negative Affect Scales (PANAS) provided summed scores for the affect measures [53]. Also included were subscales on the Purpose in Life from the Ryff Scales of Psychosocial Well-Being [54] and the instrumental support from the Piedmont Health Survey [55]. The instrumental support subscale measures both receiving help from and providing help to others. Generativity, the concern for guiding future generations, was measured through the Loyola Generativity Scale [56]. Life satisfaction was rated on a ten-point scale, modeled after the self-rated health ladder, with higher numbers representing greater satisfaction. A 4-item Likert-response format scale was used to capture home satisfaction, an index of P-E fit. 

#### 2.2.4. Demographic Variables

We controlled for demographic variables, including age (continuous), sex, and marital status (non-married versus married/partnered).

### 2.3. Statistical Methods

All analyses were performed using SPSS version 24 [57]. Basic descriptive statistics were run on measures of interest across the three housing types. One-way ANOVA tests were performed to assess mean differences for continuous and count variables. Post-hoc tests were used to identify significant mean differences between groups. Chi-square and Fisher’s exact tests were performed on categorical variables. A multinomial logistic regression model with backwards entry was fitted to examine factors associated with residence type. In this analysis, those living in the community-based housing served as the referent group. Resident profile typologies emerge from the multinomial analyses. Then, a multivariate linear regression analysis with backwards entry was used to identify demographic, health, and psychosocial factors associated with increased HCBS use. Given the large number of independent variables included in this study, backwards entry was used in multivariate analyses to generate more parsimonious and interpretable models.

## 3. Results

Table 1 provides sample characteristics by housing type. Overall, the average age of participants was 76.35 (±7.9) years. The majority of participants was female (67%) and 28% were married. Within the overall sample, self-assessed health averaged 7.40 on a ten-point scale. Health compared to others averaged 3.13 on a four-point scale, both indicative of good health. Participants averaged two limitations in activities of daily living, and had last visited their physician on average 2.20 months prior. Nearly one-quarter (24%) had been hospitalized in the two years preceding the interview for a mean average of 1.65 times for an average of ten days per stay. The three leading HCBS used by participants were senior centers (20%), homemaker services (19%), and transportation services (18%). On average, participants reported using less than one HCBS.

When comparing study characteristics by housing type, participants residing in service facilities were significantly older than community-dwelling participants. A larger proportion of those residing in service-poor facilities were female, and a significantly smaller proportion was married relative to other housing types. On average, community-dwelling older adults higher self-rated health, better ratings of health compared to others, and higher energy levels compared to the other two groups. On average, community-dwelling participants provided more instrumental support relative to those residing in facilities.

On average, community-dwelling participants reported higher life satisfaction relative to those residing in service-rich and service-poor housing. On average, feelings of generativity and having a purpose in life were lowest among service-poor participants relative to the other participant groups. On average, service-poor residents used significantly more HCBS relative to other participant groups. More specifically, a significantly larger proportion of those in service-poor facilities used home health care, transportation, home-delivered meals, and homemaker services than other participant groups. A significantly smaller proportion of service-rich residents used senior centers relative to other participant groups. A significantly smaller proportion of service-poor residents used legal services relative to other participant groups.

Table 2 presents findings from a multinomial logistic regression model that examined factors associated with residence type to generate emergent resident profiles for each housing type. Compared to community-dwelling individuals, participants who were older and married were more likely to reside in service-rich facilities. Participants with more ADLs were more likely to reside in service-rich facilities, whereas those who provided and received instrumental support were less likely to reside in service-rich facilities. Participants with higher perceived life satisfaction were less likely to reside in service-rich facilities.

Compared to community-dwelling individuals, older participants were more likely to reside in service-poor facilities. Participants who were married were less likely to reside in service-poor facilities. Participants with more ADLs were more likely to reside in service-poor facilities, whereas those who provided instrumental support were less likely to reside in service-poor facilities. Participants with higher positive affect and negative affect scores were more likely to reside in service-poor facilities; whereas those with higher perceived life satisfaction were less likely to reside in service-poor facilities.

Table 3 presents findings from a linear regression model that examined factors associated with HCBS use. Participants who resided in service-poor housing, those who were older, and females used more HCBS. Participants who had more ADLs and received more instrumental support used more HCBS. Those who provided less instrumental support used more HCBS. Participants with worse positive affect and those with higher perceived purpose in life used more HCBS.

## 4. Discussion

This study provides a glimpse into the interplay of environment, health, and service utilization among a sample of older adults. When comparing demographics, health status, and psychosocial factors by housing type, differences were observed. These findings suggest that an older adult’s environment can influence their health status, or that their health circumstance can influence where they reside (choice or by force).

Findings in this study revealed that participants in service-poor housing had higher risk in terms of health and psychosocial factors (i.e., ADLs, negative affect, life purpose). This is supported by previous research suggesting residents in senior housing have higher rates of unmanaged health needs and depression [58]. While these poorer health outcomes may hinder aging-in-place, additional research is needed to explore the underlying causes of these health indicators and the benefits of HCBS utilization. 

Overall utilization of HCBS was low among the study sample, despite reported poor health and psychosocial factors that can be addressed/improved by such services. While HCBS use was highest among those residing in service-poor housing, it is unclear whether low reported service utilization was attributed to the lack of knowledge about services, low perceived benefits from accessing services, absence of services in their local area, or service ineligibility. Findings highlight the need for additional awareness raising and recruitment efforts to promote HCBS to housing facility residents.

Providing and receiving instrumental support were associated with service use and varied across housing types. Community-dwelling older adults engaged in more instrumental support compared to facility residents. Providing less support was associated with HCBS use, while receiving more support was associated with HCBS use. Given ADLs were also associated with HCBS use, findings suggest that individuals in worse physical health may be utilizing services and resources required to meet their needs (e.g., home health care, transportation, home-delivered meals, homemaker services). Recognizing these services can be instrumental in managing health conditions and physical limitations among at-risk older adults; HCBS can be beneficial for all older adults and prevent negative health consequences. For example, because older adults’ mental health and social well-being can decline alongside growing physical limitations, and given mental health disorders are largely untreated among older adults [59,60], opportunities exist to increase mental health screening, resources, and service utilization among housing facility residents.

Based on P-E fit, study findings suggest the need to increase service coordination and build community partnerships with agencies and providers to improve fit and promote aging-in-place. For example, to combat poorer health among residents of service-poor housing, one strategy to improve health outcomes is to improve the integration of primary care and behavioral health services within housing facility communities [61]. Another strategy to improve health among housing facility residents could be to employ and work with a Health and Aging Residential Service Coordinator (HARSC), who can assess the health status of residents, determine their eligibility for services, link them to such services, and follow-up with them to ensure their needs are met [62].

In this study, the highest utilized resource was senior centers, primarily among community-dwelling and service-poor residents. Senior centers are community hubs for community-based services, especially in their offering of evidence-based programs that address health topics including chronic disease, fall prevention, and physical activity [63,64,65,66,67]. However, senior centers use and locale may limit utilization. For instance, senior centers are not widely used by diverse older adults [68], or are often located in more affluent areas. Given that transportation is among the highest reported needs for American older adults [9,69], the location of senior centers may indicate the need for transportation services among facility residents to ensure that they can access programs and resources offered at such entities. Facilities are encouraged to create partnerships with non-emergency medical transportation brokers as a strategy to increase mobility among older adults with limited travel options [70,71].

### Limitations

A limitation of this study is its cross-sectional design, thus limiting the ability to determine the causal relationships among the variables. For instance, it would be interesting to determine if living in service-poor communities actually contributes to poorer health among older adults. Second, the list of HCBS in this study may not have been comprehensive. Other services should be examined in future studies such as durable medical equipment, home safety assessments, and financial services [72]. This study is limited by the inability to delineate the actual rate of HCBS use among participants or changes in health status as a result of service utilization. Finally, the senior housing samples were convenience samples and therefore not generalizable to the greater population. Future studies should be replicated to purposively include marital dyads and use multi-level models to examine household-level service use across housing types. Study findings did not fully elucidate the factors supporting aging-in-place; future studies should empirically test the influence of the P-E framework within the context of the ecological model to include expanded environmental influences (psychosocial). Further investigation is also warranted to examine the longitudinal impacts of environment on health status among the aging population. Additionally, further investigations should examine the health-related impact on migration and relocation, which are indicators of adults’ ability to age-in-place.

## 5. Conclusions

To meet the needs of a growing aging society, improving P-E fit between older adults and their respective environments should be a priority of service providers and policy makers. When the fit between an older adult and his or her environment is insufficient and leaves health and psychosocial needs unfulfilled (e.g., needs for meal provisions, homemakers, respite care, transportation, in-home health care), it is incumbent upon service providers and policy makers to work together to improve fit to increase aging-in-place opportunities. One way to achieve this goal is by implementing interventions at multiple systems levels (e.g., individual, family, and community) that create new resources, sustain existing services, and promote health and aging-in-place.

## Figures and Tables

**Table 1:** Sample characteristics by housing type.

Variables	Total	Community-Dwelling	Service-Rich	Service-Poor	F or χ^2^	*p*
	(*n* = 663)	(*n* = 343)	(*n* = 184)	(*n* = 136)		
**Demographics**						
Age	76.35 (±7.9)	73.20 (±7.4) *	80.69 (±6.4) *	78.16 (±8.1) *	69.69	<0.001
Male	33%	39%	34%	13% *	30.28	0.010
Female	67%	61%	66%	87% *		
Married	28%	26% *	50% *	6% *	87.93	<0.001
**Health Status**						
Health Compared to Others	3.13 (±0.79)	3.36 (±0.66)	3.11 (±0.71)	3.00 (±0.93) *	3.48	0.033
Self-Rated Health	7.40 (±2.11)	8.16 (±1.67) *	7.46 (±1.90) *	7.00 (±2.37) *	4.15	0.017
ADLs (Activities of Daily Living)	2.01 (±2.76)	1.22 (±2.28) *	1.94 (±2.80) *	2.63 (±2.20) *	15.92	0.089
Energy Level	6.77 (±2.04)	7.59 (±1.61) *	6.95 (±1.56) *	5.88 (±1.64) *	6.74	0.001
Months since last physician visit	2.20 (±7.28)	1.48 (±2.23)	.94 (±1.71)	2.00 (±4.17)	2.04	0.133
Number of hospitalization (past year)	1.65 (±1.16)	1.67 (±1.02)	1.65 (±1.81)	1.50 (±0.58)	0.02	0.978
Number of days in hospital (past two years)	10.00 (±16.18)	11.80 (±19.45)	7.01 (±11.78)	8.50 (±9.68)	0.55	0.579
Provide Instrumental Support	6.00 (±2.07)	6.31 (±2.0) *	4.76 (±1.9)	4.31 (±1.6)	8.45	<0.001
Receive Instrumental Support	6.10 (±1.97)	6.22 (±2.0)	5.68 (±2.2)	5.90 (±2.0)	4.42	0.088
**Psychosocial Factors**						
Positive Affect	3.60 (±0.60)	3.67 (±0.5)	3.54 (±0.4)	3.54 (±0.9)	3.86	0.071
Negative Affect	1.61 (±0.51)	1.60 (±0.5)	1.60 (±0.4)	2.00 (±0.5)	2.94	0.055
Social Integration	3.23 (±0.41)	3.23 (±0.4)	3.14 (±0.4)	3.03 (±0.2)	12.87	0.171
Generativity	2.86 (±0.60)	3.00 (±0.5)	2.90 (±0.5)	2.50 (±0.6) *	4.12	0.018
Life Satisfaction	8.13 (±1.70)	8.70 (±1.4) *	8.20 (±1.3)	8.10 (±2.1)	3.07	0.049
Purpose in Life	5.30 (±1.33)	5.54 (±1.1)	5.33 (±1.3)	4.81 (±1.3) *	5.68	0.023
**Services Used**						
Home Health Care	5%	3%	4%	13% *	17.20	<0.001
Senior Center	20%	24%	12% *	24%	11.71	0.010
Transportation	18%	5%	28%	39% *	94.24	<0.001
Home-Delivered Meals	5%	3%	3%	15% *	32.56	<0.001
Legal Assistance	11%	5%	4%	2% *	5.81	0.010
Homemaker	19%	11%	23%	32% *	29.23	0.020
Number of Services Used	0.68 (±0.88)	0.46 (±0.72) *	0.69 (±0.82) *	1.2 (±1.0) *	41.07	<0.001

Percentages reported for categorical variables. Means and standard deviations (SD) reported for continous and count variables. * Signifies significant mean differences determined by post-hoc analyses.

**Table 2:** Multinomial logistic regression examining factors associated with housing types.

Variables	Service-Rich Facilities						Service-Poor Facilities					
	Beta	S.E.	*p*	OR	95% CI		Beta	S.E.	*p*	OR	95% CI	
					Lower	Upper					Lower	Upper
**Demographics**												
Age	0.14	0.02	<0.001	1.16	1.11	1.20	0.07	0.02	<0.001	1.07	1.03	1.11
Female	0.42	0.25	0.099	1.52	0.92	2.49	1.14	0.32	<0.001	3.13	1.67	5.87
Married	1.83	0.26	<0.001	6.20	3.74	10.27	−1.15	0.42	0.006	0.32	0.14	0.72
**Health Status**												
Health compared to others same age	0.03	0.18	0.869	1.03	0.72	1.47	0.03	0.18	0.869	1.03	0.72	1.47
Sum of ADLs	0.13	0.06	0.027	1.13	1.01	1.27	0.13	0.06	0.027	1.13	1.10	1.27
Provide Instrumental Support	−0.14	0.07	0.039	0.87	0.76	0.99	−0.25	0.07	<0.001	0.78	0.68	0.90
Receive Instrumental Support	−0.15	0.06	0.012	0.86	0.76	0.97	−0.04	0.07	0.548	0.96	0.85	1.09
**Psychosocial Factors**												
Positive Affect	0.04	0.25	0.884	1.04	0.64	1.67	0.56	0.25	0.028	1.75	1.06	2.87
Negative Affect	−0.11	0.28	0.701	0.90	0.52	1.55	0.62	0.28	0.024	1.86	1.09	3.20
Life Satisfaction	−0.19	0.08	0.022	0.83	0.70	0.97	−0.08	0.08	0.335	0.92	0.78	1.09
Purpose in Life	0.07	0.10	0.458	1.07	0.89	1.29	−0.24	0.10	0.017	0.79	0.65	0.96

Referent Group: community-dwelling individuals. Model fit statistics: Nagelkerke R^2^ = 0.49; −2Log = 951.90; χ^2^ = 348.62; df = 28; *p* < 0.001.

**Table 3:** Linear regression examining factors associated with service utilization.

Variables	Number of Services Used					
	Beta	S.E.	*t*	*p*	95% CI	
					Lower	Upper
**Service-Poor Housing vs. Other**	0.43	0.08	5.13	<0.001	0.26	0.59
**Demographics**						
Age	0.01	0.00	2.11	0.036	0.00	0.02
Female	0.20	0.07	2.94	0.003	0.07	0.33
**Health Status**						
Sum of ADLs	0.06	0.01	4.12	<0.001	0.03	0.08
Provide Instrumental Support	−0.07	0.02	−3.74	<0.001	−0.11	−0.03
Receive Instrumental Support	0.05	0.02	2.71	0.007	0.01	0.08
**Psychosocial Factors**						
Positive Affect	−0.10	0.05	−1.77	0.077	−0.20	0.01
Purpose in Life	0.04	0.03	1.72	0.085	−0.01	0.09

Adjusted R^2^ = 0.22; F (8628) = 22.84; *p* < 0.001.
